# Imaging, quantification and visualization of spatio-temporal patterning in mESC colonies under different culture conditions

**DOI:** 10.1093/bioinformatics/bts404

**Published:** 2012-09-03

**Authors:** N. Scherf, M. Herberg, K. Thierbach, T. Zerjatke, T. Kalkan, P. Humphreys, A. Smith, I. Glauche, I. Roeder

**Affiliations:** ^1^Institute for Medical Informatics and Biometry, Medical Faculty Carl Gustav Carus, Dresden University of Technology, Fetscherstrasse 74, D-01307 Dresden, Germany; ^2^Interdisciplinary Centre for Bioinformatics, University of Leipzig, Haertelstrasse 16-18, D-04103 Leipzig, Germany; ^3^Wellcome Trust Centre for Stem Cell Research (Stem Cell Institute), University of Cambridge, Tennis Court Road, Cambridge CB2 1QR, UK

## Abstract

**Motivation:** Mouse embryonic stem cells (mESCs) have developed into a prime system to study the regulation of pluripotency in stable cell lines. It is well recognized that different, established protocols for the maintenance of mESC pluripotency support morphologically and functionally different cell cultures. However, it is unclear how characteristic properties of cell colonies develop over time and how they are re-established after cell passage depending on the culture conditions. Furthermore, it appears that cell colonies have an internal structure with respect to cell size, marker expression or biomechanical properties, which is not sufficiently understood. The analysis of these phenotypic properties is essential for a comprehensive understanding of mESC development and ultimately requires a bioinformatics approach to guarantee reproducibility and high-throughput data analysis.

**Results:** We developed an automated image analysis and colony tracking framework to obtain an objective and reproducible quantification of structural properties of cell colonies as they evolve in space and time. In particular, we established a method that quantifies changes in colony shape and (internal) motion using fluid image registration and image segmentation. The methodology also allows to robustly track motion, splitting and merging of colonies over a sequence of images. Our results provide a first quantitative assessment of temporal mESC colony formation and estimates of structural differences between colony growth under different culture conditions. Furthermore, we provide a stream-based visualization of structural features of individual colonies over time for the whole experiment, facilitating visual comprehension of differences between experimental conditions. Thus, the presented method establishes the basis for the model-based analysis of mESC colony development. It can be easily extended to integrate further functional information using fluorescence signals and differentiation markers.

**Availability:** The analysis tool is implemented C++ and Mathematica 8.0 (Wolfram Research Inc., Champaign, IL, USA). The tool is freely available from the authors. We will also provide the source code upon request.

**Contact:** nico.scherf@tu-dresden.de

## 1 INTRODUCTION

Mouse embryonic stem cells (mESCs) are derived from the inner cell mass of a blastocyst-stage embryo. Under appropriate conditions these cells can be maintained in a proliferative and undifferentiated state over many passages (self-renewal) while retaining the ability to differentiate into a multitude of different cell types and repopulate an embryo (pluripotency) (Evans and Kaufman, 1981; Smith, 2001).

Depending on the culture conditions, mESCs reveal different types and levels of heterogeneity. Conventional culture conditions that promote self-renewal contain the cytokine leukemia inhibitory factor (LIF) and serum factors. However, it has been demonstrated that mESCs in these conditions show substantial variations in the expression levels of the transcription factors (TFs) Nanog and Rex1 associated with the differentiation propensity of the cells (Chambers *et al.*, 2007; Toyooka *et al.*, 2008). Replacing LIF/serum conditions by 2i media, a novel serum-free medium containing two small inhibitor molecules, mESCs are captured in a pluripotent ground state without any spontaneous differentiation (Ying *et al.*, 2008). TF variations are greatly reduced and a rather stable and homogenous population of mESCs is achieved.

Interestingly, it appears that differences in the state of a cell are not only detectable on a molecular level, but are also reflected in the *morphology* and the *spatial arrangement* of single mESCs and the resulting cell colonies. Although mESCs cultured in 2i media form homogenous, dense clusters of cells, the same cells spread out under LIF/serum forming rather flat and spatially extended cell colonies.

In order to study the spatio-temporal behavior of mESCs, it is ultimately necessary to establish a framework allowing for the ‘quantification’ of time-dependent properties of cells and cell colonies. To systematically analyze these morphological differences with respect to colony growth, shape, motion, dynamic pattern formation and structural homogeneity, we established a live-cell imaging system to continuously monitor mESC colonies under defined conditions. In the present work, we introduce a novel bioinformatics approach to automatically quantify the spatio-temporal structure of such cell cultures based on time-lapse image sequences. Herein, we focus on the description of the image-analytical methodology and show a set of potential measures and visualizations for the quantification of temporal colony development. We use a preliminary dataset of mESC cultures to demonstrate the feasibility of our approach and to discuss further applications. In particular, we argue that such quantitative measures are a fundamental prerequisite for the establishment of computational models of tissue organization.

## 2 APPROACH

Advances in live-cell imaging techniques and long-term cell cultures provide an opportunity to study the spatial arrangement of mESCs as well as dynamical pattern formation within cell colonies. Since manual analysis of image sequences is time-consuming, tedious and suffers from intra- and inter-observer differences, we developed an automated method that allows for a long-term tracking of mESCs cultures in defined conditions. A number of methods have been proposed for tracking of *individual cells* (see e.g. Acton and Ray (2006) and Meijering *et al*. (2009) for an overview). However, these techniques are not directly applicable to the analysis of cell colonies in a straightforward manner. Since individual cells cannot be visually separated (in particular for the spherical colonies in 2i medium) and the fact that cell clusters show characteristics that differ from those of single cells (e.g. frequently occurring fusion of cell clusters, large inhomogeneities in object structure and motion, drastic changes in appearance) a more general method for tracking of deformable tissue structures over time is needed. The direct tracking of single cells inside a colony is not possible, consequently, an analysis of the overall changes in the images of two consecutive time-points (on a single pixel level) is required to describe the dynamical changes inside a colony. This method allows for a quantification of the *dynamic internal structure* of colonies on different levels of description (sub-cellular structures, cells, cell ensembles, etc.) For this purpose, we extend our previously published method for single cell tracking based on nonparametric image registration (Scherf *et al.*, 2012). In particular, our approach is based on a fluid image registration to estimate dense deformation fields describing the displacement of structures between consecutive images. We decided to use a fluid-like model as it seems appropriate to describe the shape changes of cells and cell ensembles and, from a more technical point of view, allows for the mapping of rather large changes between consecutive images as opposed to e.g. elastic models (cf. Bro-Nielsen and Gramkow, 1996; Fischer and Modersitzki, 2004). The classical fluid registration model (Bro-Nielsen and Gramkow, 1996) has also been used by Hand *et al*. (2009) and Tokuhisa and Kaneko (2010) in the context of single-cell tracking. However, in contrast to these works our registration approach is able to handle partial fusion of tracked objects, which is crucial for tracking of colonies, that constantly merge, absorb and emit cells over time. Furthermore, our method is based on a variational formulation as introduced by Kuska *et al*. 2008, which allows greater flexibility in handling different matching criteria and regularization constraints.

**Fig. 1.**
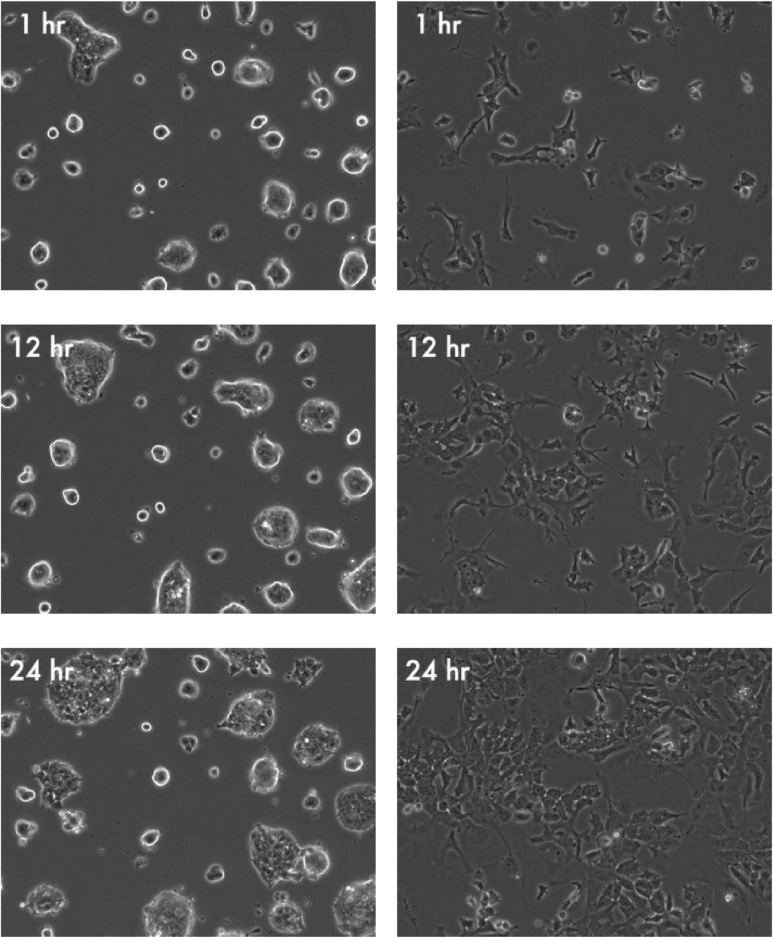
Image data. Phase contrast images from the sequence of mESC colonies cultured under 2i conditions (left column) and LIF/serum conditions (right column) at three different time points

We performed an initial set of experiments to establish a reference dataset for the quantification of colony development. In particular, we obtained sequences of time-lapse microscopy images (phase contrast images) for a Rex1-GFPd2 mESC line maintained under 2i and LIF/serum conditions. Cells were monitored for 24 h with a frequency of one image per hour (Fig. 1). We started out by quantifying global and colony-specific parameters (e.g. size, shape and internal structure) as functions of time in order to *morphologically* characterize mESC development in different conditions promoting self-renewal. Furthermore, we used the obtained estimates of dynamical changes between images from the fluid image registration to quantify *dynamic structural properties* of the colonies (mean value, and standard deviation of internal motion vectors). Moreover, the heterogeneity of these measures within and between different culture conditions is analyzed. Finally, we reconstructed the complete spatio-temporal growth patterns to visualize spatial spread, merging and splitting of the initial colonies and dynamic changes in their structural properties to facilitate visual comprehension of the different experiments.

## 3 METHODS

### 3.1 Cell cultures

Mouse embryonic stem cells were cultured without feeders in the presence of LIF in GMEM containing 10% fetal calf serum or in serum-free N2B27 supplemented with MEK inhibitor PD0325901 (1 *μ*M) and GSK3 inhibitor CH99021 (3 *μ*M), together known as 2i (Ying *et al.*, 2008).

### 3.2 Microscopy

A Nikon BioStation IM-Q with a cell incubator, a motorized inverted microscope and a high-sensitivity CCD camera was used to image the colonies over time by parallel acquisition of both phase contrast and fluorescence images of living mES cells (only the phase contrast images were used for the present study). Images of mESC colonies are taken over 24 h with a frequency of one image per hour at a magnification of 10×. Digital images with a size of 640×480 pixels were obtained in uncompressed TIF format for subsequent processing.

### 3.3 Image analysis

#### 3.3.1 Fluid-like image registration

Given a (deformable) *source* image 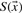 and a (fixed) *template* image 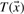 , non-parametric image registration aims at finding a displacement field 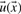 that maps 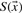 onto 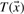 such that 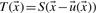 , i.e. that maps each pixel of *S* to the corresponding position in image *T*, such that the gray level intensity of both images match at each pixels position (typically in the least squares sense). This formulation of the problem is known to be ill-posed (Horn and Schunck, 1981), thus requiring additional constraints on the properties of the transformation to yield a unique solution. Our regularization is based upon the curvature constraint introduced by Fischer and Modersitzki (2004), since it exhibits a number of interesting properties. However, the dynamics of fluids is better described in terms of the *velocity field* 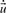 rather than in terms of the displacement field 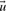 itself. Consequently, the regularization is applied to 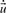 to yield a fluid model. This idea has been introduced by Kuska *et al*. (2008). We briefly derive the corresponding formulae for this model in the following.

As stated above, we are interested in a displacement field *u* that minimizes the following Lagrangian functional (we use an Eulerian reference frame for description of the displacement):
(1)
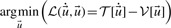

with
(2)
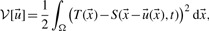

describing the matching of the gray values between the template and the deformed source image and
(3)
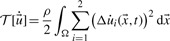

for the regularity of the underlying velocity field. The necessary condition for a displacement field 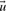 to be a minimizer of Equation (1) can be obtained by the derived Euler–Lagrange equation yielding:
(4)
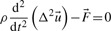

with 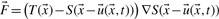 . This equation describes an undamped motion since the Lagrangian ℒ is free of dissipation of energy. To prevent oscillations in the solution, artificial damping is introduced to enforce convergence when both images match as closely as possible. By using a position independent damping 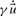 and a viscosity term 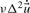 and formally taking the over-damped limit, one arrives at the final formulation:
(5)
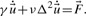

For further details regarding the formal derivation, we refer to Kuska *et al*. (2008). The resulting partial differential equation has to be solved numerically to obtain the displacement field 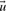 . Here, we use a fast Poisson solver to handle the biharmonic operator in space and step-size controlled Runge–Kutta method (Verner, 1993) for the time dependent part. The method is implemented in C++ and parallelized using OpenMP and Threading Building Blocks (TBB) library.

**Fig. 2.**
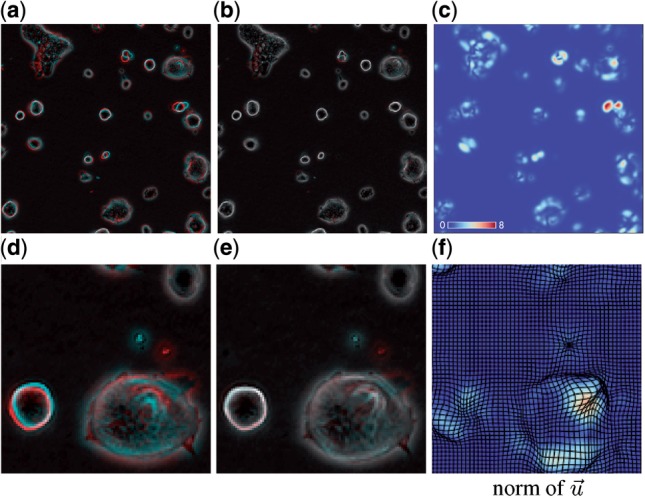
Fluid-like image registration. Example showing the result of the fluid registration for a sample of the 2i sequence. Shown is the mismatch before **(a)** and after **(b)** registration. Unmatched structures between two consecutive images show up as colored regions (cyan and red) whereas gray areas correspond to areas with matching gray values. The magnitude of the vectors of the resulting displacement field are visualized in **(c)**, see inset for scaling of color function. (d-f) show a zoomed subpart of the image

The first row of Figure 2 shows the results of a registration of two images from the 2i sequence (a zoomed subpart of the image is shown in the second row). The mismatch between the images before (a) and after (b) registration is visualized by overlaying the images in different color channels. Non-matching pixel values between images are visible as colored regions (red and cyan) while matching pixel values appear as gray values. Panel (c) shows a plot of the magnitude of the corresponding displacement field (magnitude is color-coded on a color scale from blue—0 pixel displacement to red—up to ca. 8 pixel displacement). A deformed grid is further shown as an overlay in the zoomed part to visualize the displacement field more clearly. The resulting displacement field is further used to estimate the motion within colonies between two consecutive observations.

#### 3.3.2 Image segmentation

**Fig. 3.**
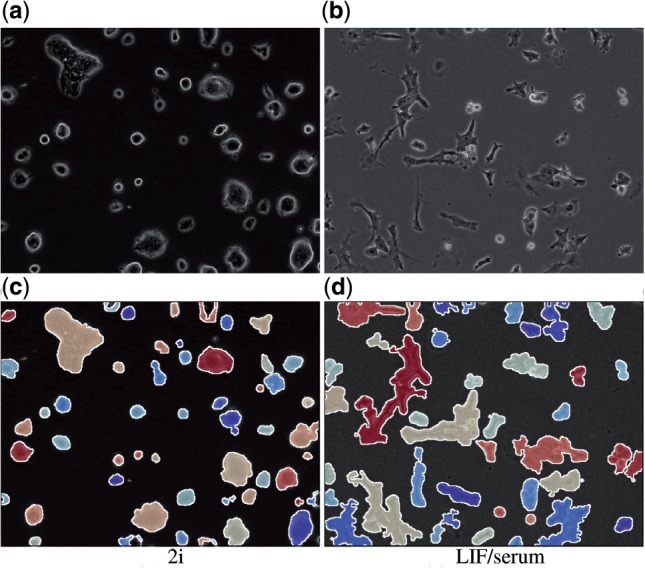
Segmentation of colonies. Example results of automated detection of individual cell colonies for **(c)** 2i and **(d)** LIF/serum conditions. Colonies are colored randomly. (a) and (b) show the unprocessed images for comparison

To handle typical problems with phase-contrast images (e.g. halos around cells and inhomogeneities of signal intensity) we use a retinex filter to locally adapt the gray values of the image (Land and McCann, 1971). Next, total variation filtering is applied to reduce noise while preserving important image structures (Chan *et al.*, 2001). The colonies are subsequently detected by using the active contour method proposed by Chan and Vese (2001). Finally, we apply a filling transform to close internal holes in the segmented colony structures. All these steps are implemented by the standard methods supplied with Mathematica 8.0 (Wolfram Research Inc., Champaign, IL, USA). Figure 3 shows a visualization of the results of the colony detection for 2i (c) and LIF/serum (d) conditions, respectively. (The corresponding original images are depicted in the upper row (a, b).)

#### 3.3.3 Colony tracking

For the tracking of colony development, we extended the method recently introduced in Scherf *et al.* (2012). Briefly, the initial mask of objects is obtained by a segmentation of the first image of the sequence. We use the automated segmentation described above to detect the colony shapes. The algorithm then proceeds by iterating the following steps:

- At first, the displacement between images *t* and *t* +1 is obtained by the fluid registration described above. The label masks *ℒ_t_* containing the detected and labeled objects from time *t* are then propagated to time *t* +1 by applying the resulting displacement field 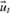 to the label mask resulting in an estimate of new label positions 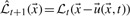.
- These masks are used as an initialization for the active contour segmentation (see above) to obtain a second label mask 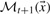 of the actual image data at *t* +1.
- Then both masks, 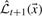 (obtained by propagated labels from *t*) and the newly segmented 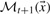 are fused to achieve a consistent labeling for the current time point. In particular, the following cases are considered:
  **–** *one-to-one matching* between 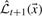 and 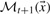 In this case, the segmented mask 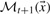 is used, and the label is taken from the propagated 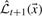

  **–** *merging of objects*: Here, 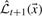 predicts a number of separated objects, where only one single cluster could be detected by the segmentation. In this case, the propagated masks of 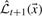 are kept and the segmentation result is discarded.
  **–** *splitting of objects*: Since the fluid registration typically preserves the topology of the masks, a connected mask will be predicted from the deformation in 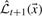 even if a colony splits (or a cell is separated from the colony). On the other hand, the segmentation 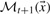 will provide a number of separated objects. In this case, the masks from 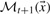 are used and new labels for the substructures are introduced (indicating *clonal* inheritance of subcolonies or cells).
  **–** *newly occurring objects*: To handle objects entering the field of view or to start a new track if the old one has been lost, we simply provide new labels in 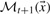 for objects that have not received a label from 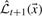 by the propagation process.

These steps are then repeated for each time point. The method needs 30~40 s to process one frame, where most of the time is needed to calculate the fluid registration. The speed can be optimized at the cost of registration quality if needed. For the present study of colony development, we applied the tracking method in a backward fashion by reversing the image sequence. This is advantageous since the handling of object splitting results in better masks (obtained from segmentation) than in the case of object fusion (where one has to rely on the masks deformed by the registration step) and counteracts deterioration of the masks over time. If image analysis should be done in *real-time* during image acquisition, the method can also be applied in the usual forward manner. A validation of the method regarding its performance for automated single cell tracking was reported in Scherf *et al.* (2012) for several experimental and synthetic data sets.

### 3.4 Growth statistics

To analyze a wide range of structural properties of colony growth in space and time, we determine the following measurements describing certain aspects of mESC cell colonies.

#### 3.4.1 Global measurements of colony growth

The *total (surface-) area* covered by all cell colonies over time is the simplest measure to address overall colony growth. This measure is obtained by dividing the total area covered by detected cells and cell colonies by the total image size (in pixel) for each frame.

#### 3.4.2 Shape parameters of individual cell colonies

In order to obtain a more detailed analysis of the morphological differences between cell cultures and to address the variability of spatial structures over time, we calculate the following features for each individual colony at each time point:

- the area covered by the colony (counted in image pixels)
- the elongation of the colony: *ξ* = 1 – λ_1_*/*λ_2_ where λ_1_ and λ_2_ are the major and minor axis of an ellipse that best matches the shape of the colony. This provides an estimate of the roundness (*ξ* ~ 0) or elongation (*ξ* ~ 1) of the corresponding colony.
- the circularity of the colony defined as: *γ* = 4*π a/p*^2^, where *a* denotes the area and *p* the perimeter of the object, respectively. This ratio equals one for a circle and approaches zero for structures of increasing irregularity.

#### 3.4.3 Structural parameters within cell colonies

Cell colonies show an inherent level of heterogeneity, e.g. with respect to cellular orientation, brightness, etc. In order to quantify these structural characteristics, we propose the following quantity:

- the entropy of the image values quantifies the level of spatial inhomogeneities within a particular colonies. Precisely, we calculated the base *e* entropy of the respective gray values within the image region inside colony *j*, i.e. *H_j_* = – ∑*_i_p*(*g_i_*)log(*p*(*g_i_*)) where the sum is taken over each possible gray level *g_i_*. The samples are taken at spatial positions 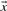 if 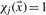 , where *χ_j_* is the characteristic function for the mask *j*.

#### 3.4.4 Dynamical changes within cell colonies

The displacement fields obtained by the registration of subsequent images facilitate an analysis of the dynamic changes within each individual colony. For this purpose, we analyze the region of the displacement field lying inside the respective colonies (*χ_j_* ( 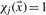 , see above) from one time point to the next. In particular, the following measures appear most suitable (in light of subsequent mechanistic modeling):

- the *mean displacement comprises* the dynamic changes (caused by motion of cells within the cell clusters) in the colony. It is calculated over the norm of the displacement field vectors within the respective area covered by the colony.
- the *standard deviation of the displacement* addresses heterogeneity of the displacement field vectors within the respective colony.

Both measures address dynamical changes of individual colonies between subsequent image frames in a holistic manner. Thus, they have the advantage of being applicable even if no single cell measurements are directly available.

### 3.5 Visualization of spatio-temporal patterns of colony growth

The illustrated tracking method allows us to follow individual colony development over multiple images. Akin to single cell tracking, we use a kind of genealogical information to generate novel visualizations of colony growth in space and time. Topological changes in the population and the dynamics of each individual colony are depicted by means of a stream-like metaphor. The general principle is the following: individual colonies are placed horizontally where the one-dimensional extension is plotted proportional to a chosen feature (here the area covered by the circularity). A color-coding visualizes the state of the colony with respect to a second feature (here the circularity). The temporal dimension is plotted in vertical direction pointing downwards, i.e. the dynamic changes of each colony are visualized as a downward stream. Merging and/or splitting of colonies is visualized by converging or diverging streams. Taken together, we can illustrate how the individual colonies grow, deform, merge and split over time. Since all colonies can be displayed in parallel, the population nexus is preserved for each individual colony (i.e. its relation to the whole population at time *t*). A representative example is provided below in Figure 5.

## 4 RESULTS AND DISCUSSION

We have established a novel algorithm for the identification, quantification and temporal tracking of mESC colonies. In the following, we show the general applicability of the method and use a selection of the measures (as introduced above) to demonstrate the descriptive power of our approach. In particular, we rely on a preliminary dataset obtained for self-renewing mESCs cultured either under 2i or LIF/serum conditions. We did preliminary statistical tests on the pooled data (over temporal dimension) to test for differences in the median of the distribution of the respective features between both conditions. A non-parametric Wilcoxon rank sum test showed differences between the conditions for all proposed features at a level of significance below 0.01. However, it should be pointed out that the primary aim was to test the applicability of features that are meaningful in the context of further spatial, mathematical modeling approaches rather than pure classification. A number of obtained results are discussed in the following.

**Fig. 4.**
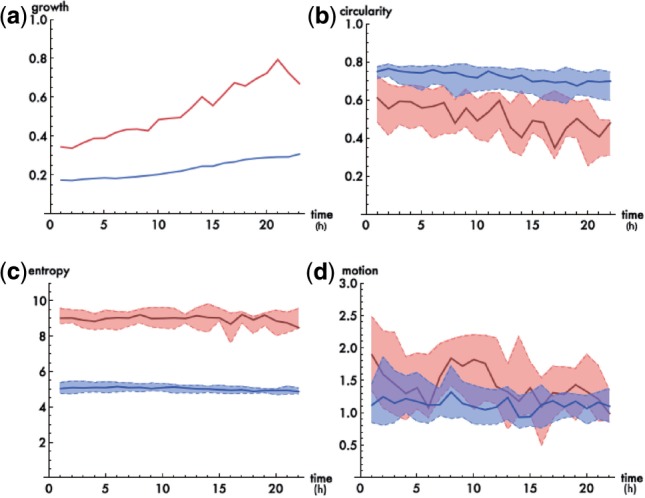
Application of structural measures for colony development. **(a)** overall colony growth measured as the fraction of covered surface (2i—blue, LIF/serum—red) over time (h), **(b–d)** plots illustrating the distribution (median as thick line and respective 0.25 and 0.75 quantiles as dashed lines) of colony-based measures for each image frame in chronological order [(b) -circularity, (c) - entropy, (d) - mean displacement]

Figure 4a illustrates overall growth curves for both conditions (2i- blue, LIF/serum - red). The graphical representation supports and verifies the naive impression that mESCs in LIF/serum tend to grow in more flattened, space-consuming monolayers. After 24 h almost the whole surface area is covered by cells under LIF/serum conditions whereas for 2i cells, the covered area increases only slightly. This effect indicates underlying differences in the proportion of cell–cell to cell-surface adhesion in 2i as compared to LIF/serum, leading to differences in colony spreading and shape.

**Fig. 5.**
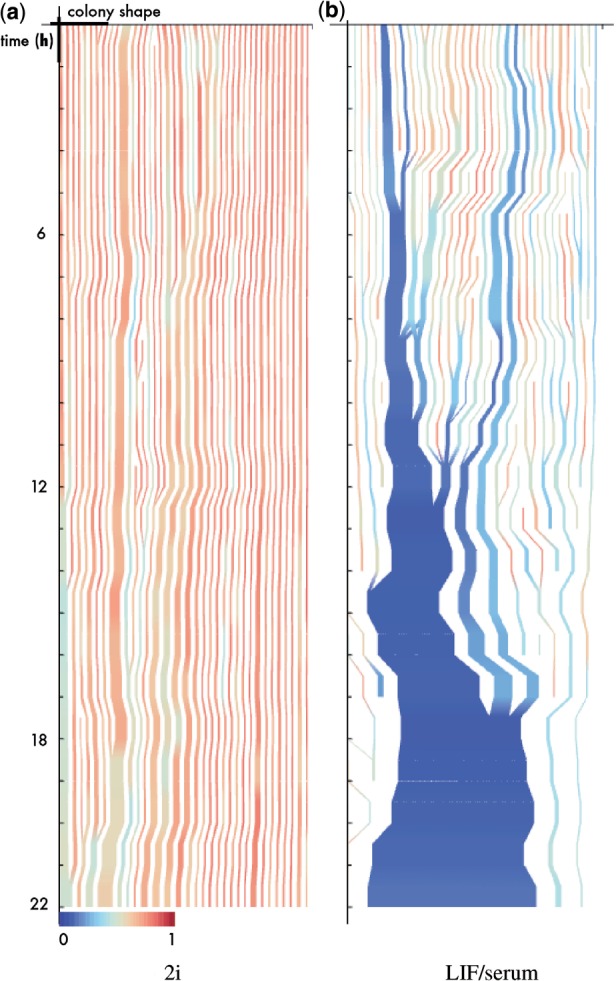
Stream-like visualization of colony development. The frame number is given on the *y*-axis, while the thickness of lines corresponds to area of colony. Color coding is according to circularity (red—round, blue—irregular; color scale is given below the plot). Merging and branching of streams depicts merging and splitting of colonies. Width of plot is normalized with respect to complete area of the observed region (a value of 1 would correspond to complete confluency)

As an example for the shape parameter of individual cell colonies, we show a plot of the median (thick line and respective 0.25 and 0.75 quantiles as dashed lines) of the distribution of *colony circularity* in Figure 4b. Although a direct comparison for the individual time points is not in place given the initial differences, e.g. in local cell densities, it appears evident that colonies under 2i are more regularly shaped (round) as compared to more diverse and complicated structures found in colonies under LIF/serum. This observation supports the visual impression obtained in Figure 1 and is further visualized in Figure 5.

Furthermore, we analyzed the homogeneity of the gray levels within the colonies under both conditions using the outlined entropy measure. Figure 4(c) indicates that 2i colonies are much more homogeneous as compared to colonies under LIF/serum. These differences in the phase contrast images most likely derive from differences in the cell–cell and cell–substrate adhesion as well as the cell shape. Cells cultured under LIF/serum conditions are more attached to the surface and less adhesive to each other leading to a far more heterogeneous distribution of grey levels since more individual cells and sub-cellular structures are visible. 2i cells, on the other hand, form rather compact, three-dimensional spheres.

As an example addressing the dynamical changes within cell colonies we show the distribution of the *mean displacement* between subsequent images in Figure 4d. It appears that colonies under LIF/serum conditions have an increased dynamical remodeling ability. The plot indicates that there is a higher tendency to find colonies with larger displacements under LIF/serum as compared to 2i conditions. However, a rigorous statistical analysis of these results requires a more solid and extensive dataset.

Finally, we present an example of our stream-like visualization introduced above, to demonstrate its use for explorative data analysis. Figure 5 shows the resulting plots, where the area of the individual colonies is used to code the width of the lines and the color indicates one of the shape parameters (circularity) of the colony at the respective time point. A reddish coloring corresponds to a round colony shape and blue to irregular structures. This visualization clearly highlights the structural differences between both conditions. Both experiments start from a comparable distribution of colonies (with respect to size and shape). Although the growth and the topological changes (merging, splitting) of colonies is rather homogeneous under 2i, the picture radically differs under LIF/serum conditions. Here, the initial cell distribution soon spreads out over time (note the variations in size and color) in a less ordered fashion, with an emerging large cluster and scattered smaller parts.

## 5 CONCLUSION

We have established a sophisticated framework for the automated image analysis, quantification and visualization of spatio-temporal patterning in mESC colonies. Apart from standard image analysis methods, our approach uses a fluid-like image registration to assess the temporal development of cell colonies and to extract the developmental history of individual cell colonies. This approach is applicable at different levels of granularity (individual cells and clusters of cells), depending on the underlying image data.

Applying this methodology to a preliminary dataset, we demonstrate in a proof-of-principle that mESCs show a distinctly different growth pattern under 2i and LIF/serum conditions, which are both used to maintain stem cell pluripotency. Although our analysis was initially focused only on a rather short sequence of phase contrast images, we can deduce that mESCs under LIF/serum conditions are more volatile and more heterogeneous as compared to culturing these cells under 2i conditions. These initial results demonstrate the feasibility of our approach and call for analysis of further image sequences to improve statistical power. It also appears very interesting to compare the results to growth patterns under other different seeding densities (e.g. starting from clonal density) and higher image frequencies. As the next step, we will establish a thorough benchmark for our system by analyzing a larger number of experiments under different conditions and create manually validated reference data. We will further explore more complex measures of colony shape and structures, e.g. Zernike- or Fourier decompositions and PCA or ICA as discussed in Pincus and Theriot (2007).

Our approach establishes the general framework to analyze colony development of mESCs under various conditions. The approach can be extended to incorporate further available information as it is contained in fluorescence images or under differentiation inducing conditions. This will reveal possible correlations between internal cell states and spatio-temporal structure of colonies. In the long run, we aim to use this data to develop a mathematical model of mESC organization representing both intra-cellular and inter-cellular regulations. In particular, we will extend a previous established TF network model (Glauche *et al.*, 2010) by a spatial dimension, thus including the concept of spatial heterogeneity among mESCs. The presented methods for tracking, analyzing and visualizing the structural features of cell colonies over time will be indispensable to bridge the gap between phenomenological measurements and mechanistic principles in the context of mathematical modeling approaches.
